# Effects of a Web-Based Intervention for Stress Reduction in Primary Care: A Cluster Randomized Controlled Trial

**DOI:** 10.2196/jmir.4246

**Published:** 2016-02-12

**Authors:** Michael Mehring, Max Haag, Klaus Linde, Stefan Wagenpfeil, Antonius Schneider

**Affiliations:** ^1^ Institute of General Practice Klinikum rechts der Isar Technische Universität München Munich Germany; ^2^ Institute for Medical Biometry, Epidemiology und Medical Informatics (IMBEI) Universitätsklinikum des Saarlandes Homburg/Saar Germany

**Keywords:** Web-based, randomized controlled trial, life stress, stress reduction

## Abstract

**Background:**

Preliminary findings suggest that Web-based interventions may be effective in achieving significant stress reduction. To date, there are no findings available for primary care patients. This is the first study that investigates a Web-based intervention for stress reduction in primary care.

**Objective:**

The aim was to examine the short-term effectiveness of a fully automated Web-based coaching program regarding stress reduction in a primary care setting.

**Methods:**

The study was an unblinded cluster randomized trial with an observation period of 12 weeks. Individuals recruited by general practitioners randomized to the intervention group participated in a Web-based coaching program based on education, motivation, exercise guidance, daily text message reminders, and weekly feedback through the Internet. All components of the program were fully automated. Participants in the control group received usual care and advice from their practitioner without the Web-based coaching program. The main outcome was change in the Perceived Stress Questionnaire (PSQ) over 12 weeks.

**Results:**

A total of 93 participants (40 in intervention group, 53 in control group) were recruited into the study. For 25 participants from the intervention group and 49 participants from the control group, PSQ scores at baseline and 12 weeks were available. In the intention-to-treat analysis, the PSQ score decreased by mean 8.2 (SD 12.7) in the intervention group and by mean 12.6 (SD 14.7) in the control group. There was no significant difference identified between the groups (mean difference –4.5, 95% CI –10.2 to 1.3, *P*=.13).

**Conclusions:**

This trial could not show that the tested Web-based intervention was effective for reducing stress compared to usual care. The limited statistical power and the high dropout rate may have reduced the study’s ability to detect significant differences between the groups. Further randomized controlled trials are needed with larger populations to investigate the long-term outcome as well as the contents of usual primary care.

**Trial Registration:**

German Clinical Trials Register DRKS00003067; http://drks-neu.uniklinik-freiburg.de/drks_web/navigate.do?=DRKS00003067 (Archived by WebCite at http://www.webcitation.org/6eXk0PXmO)

## Introduction

Nowadays, almost all people worldwide experience increased stress. In the last few years, many studies have found an enormous increase of stress in adults, teenagers, and children [1]. Especially in Western countries, the rise in workload has resulted in a rapid growth of the number of employees experiencing psychological problems related to occupational stress [2]. In 2006, an international survey revealed that approximately 75% of the general population in developed countries reported stress on a daily basis. In addition, 44% of Americans surveyed in 2010 specified that they had experienced a growth in stress over the past 5 years [3].

There are few findings known for a Web-based intervention in primary care because most eHealth interventions for stress have been evaluated in workplace settings. Stress could be perceived as such a minor problem that it does not require any treatment or professional assistance [4]. However, there is no doubt that chronic stress clearly is a risk factor for a wide range of mental and physical health problems, such as metabolic syndrome [5], diabetes [6], cardiovascular disease [7,8], ischemic stroke [9], and depression [10-12]. Internet-based interventions have shown to be effective in community and clinical settings, including the treatment of depression [13-16], sleep disorders [17], weight reduction [18], smoking cessation [19], and stress reduction [20-27]. Some studies have also failed to find any effects on stress [28-30]. A meta-analysis showed that cognitive behavioral interventions are more effective in stress reduction than other techniques, such as relaxation techniques, multimodal programs, and organization-focused interventions [31]. Additionally, it has been noted on the basis of several trials that the effect sizes from Internet-based stress management programs were close to estimations of face-to-face cognitive behavioral interventions [31,32]. To use health care resources at an optimal level, graded treatment systems represent attempts to improve the efficiency and access to mental health. In a first attempt, low-cost interventions are offered. For those who are not sufficiently helped by the initial low-cost intervention, more intensive and costly interventions are then used in a second step [33]. In addition to the well-established intensive and costly interventions [34], the need to implement and to verify interventions with low financial and accessibility thresholds is still demanded [35,36]. Therefore, a Web-based program was developed that combines an individually tailored strategy for stress reduction with automated advice and feedback elements based on cognitive behavioral therapy (see Multimedia Appendices 1 and 2). In the cluster randomized trial reported subsequently, we investigated whether adult primary care patients who wanted stress reduction and used a fully automated 12-week Web-based coaching program did reduce their stress more effectively than with usual care by general practitioners (GPs).

## Methods

### Design

The study was designed as a 2-arm, unblinded, cluster randomized controlled trial. At the beginning of the study, approximately 2000 Bavarian GPs received a fax by the Bavarian Association of General Practitioners with information about the research project. The only inclusion criteria for GPs were interest in participating in the study and Internet access within their practice. All interested GPs were sequentially registered for randomization. After giving written consent, the participating GPs were randomized to either the interventional or the control arm. The sequence of randomization used (cluster allocation ratio 1:1) was provided by a methodologist, who did not participate in the execution of the study, via the program Research Randomizer [37]. Randomization was concealed by using sequentially numbered, opaque, sealed envelopes held by the study coordinator. Randomization was performed on the cluster level for logistical reasons (less complicated informed consent, only one intervention per doctor’s practice, limited resources requiring less training visits). Before starting the recruitment of patients, physicians received detailed instructions from the research team on the study process (both intervention and control group) and on the coaching program (only intervention group). Physicians in both groups received a detailed introduction with all study documents by post. A separate visit of all participating physicians in the intervention group took place afterwards to instruct them about the Web-based intervention with the help of case studies and to eliminate ambiguities on site with all involved GPs and the participating medical staff. Physicians assigned to the control arm were asked to change nothing in their usual way of counseling and to treat participants in the same manner as if they would have been nonparticipants. There was no structured documentation of the care provided. The patients recruited by physicians for the intervention received free access to the Web-based coaching program. The patients in the control arm were advised by the GPs in their individual way of usual measures to reduce stress. The study was approved by the Medical Ethics Committee of the Technische Universität München (April 19, 2011) and was in accordance with ethical standards for human experimentation established by the Declaration of Helsinki. All participants gave written informed consent. A data and safety monitoring board was established before the beginning of the study. The study was registered on the German Clinical Trials Register (registration number: DRKS00003067). The CONSORT eHealth checklist is shown in Multimedia Appendix 3.

### Participants and Procedures

Participating physicians were GPs in Bavaria, Germany. The GPs were requested to recruit individuals with a desire for stress reduction. Individuals who were at least 18 years of age and had Internet access were potentially eligible. GPs were asked to exclude individuals younger than 18 years, with insufficient German language skills, who did not have Internet access, suffered from a psychiatric disorder, or had a psychiatric disorder documented in the past.

After the GP decided that the patient was recommendable to participate, an information form was given and discussed with the patients and a participation form had to be signed. At the same time, baseline data acquisition took place. All participants were asked to fill in a standardized questionnaire with the GP. The standardized questionnaire consisted of the following information: age, sex, height, weight, family status, physical activity level, and the Perceived Stress Questionnaire (PSQ). The PSQ assesses subjectively experienced stress independent of a specific and objective occasion; therefore, it can be widely used without the restrictions based on age, gender, or profession. This instrument is particularly of interest if perceived stress has to be asked directly without inferring it from control or coping appraisals. In addition to providing an overall score, it also provides scores on different facets of perceived stress, such as worry, tension, joy, and demands. Participants of the intervention group received a password to the webpage, which allowed free access to the program. Participants in both groups were requested to document the follow-up evaluation together with their physician after 12 weeks. The follow-up was comprised of a repeated PSQ and information about possible adverse events. Physicians in the intervention and control group received €25 per participant for time and effort. Participants in the intervention group received free access to the stress-reduction program, which would usually cost €49. Participants in the control group received €10 as an incentive to come to their doctor’s practice for the follow-up investigation after 12 weeks. All physicians could contact the study coordinator by phone or email at any time. During the trial, a status survey was carried out on a regular basis every 6 to 8 weeks to check the number of enrolled patients and to remind about pending follow-ups. These calls were also used to solve any problems that had occurred. In addition, every 6 to 8 weeks written feedback about the number and status of participants was sent to the GPs to ensure a smooth process of the trial. No methodological changes were made during the entire study period.

### Intervention

A specific website was developed for the participants to allow log-ins without charge [38]. After completion of a preassessment, the program generated a personalized coaching program based on the participants’ physical characteristics and their everyday behavior. The coaching program was based on the generally accepted principles of cognitive behavioral therapy and combined psychoeducation and motivational techniques with behavioral therapeutic elements [39]. The content of the coaching program aimed at achieving a lasting change of behavioral patterns with the help of individualized education, motivation, exercise guidance, daily text message reminders, and self-monitoring via the Internet. The framework of the program was based on the idea by Oetting [40]. The intervention was exclusively Web-based and was not integrated into the practice system. The development and operation of the Web-based stress-reduction program was carried out by WeCARE GmbH, Göttingen, Germany. The coaching program was subdivided into 12 different constitutive modules. The module learning objectives were:

1. Being strong against stress
2. Your personal stress profile
3. Your personal stress patterns
4. Your path to more calmness
5. Release tension and recharge
6. Stress caused by grief
7. Be strong—even without others
8. Components of balance
9. Stress—the knight in shining armor
10. Stress-free—even in the workplace
11. Active against the pressure
12. Find peace and relaxation
13. On the way to relief
14. Now you are your own coach

**Figure 1:**
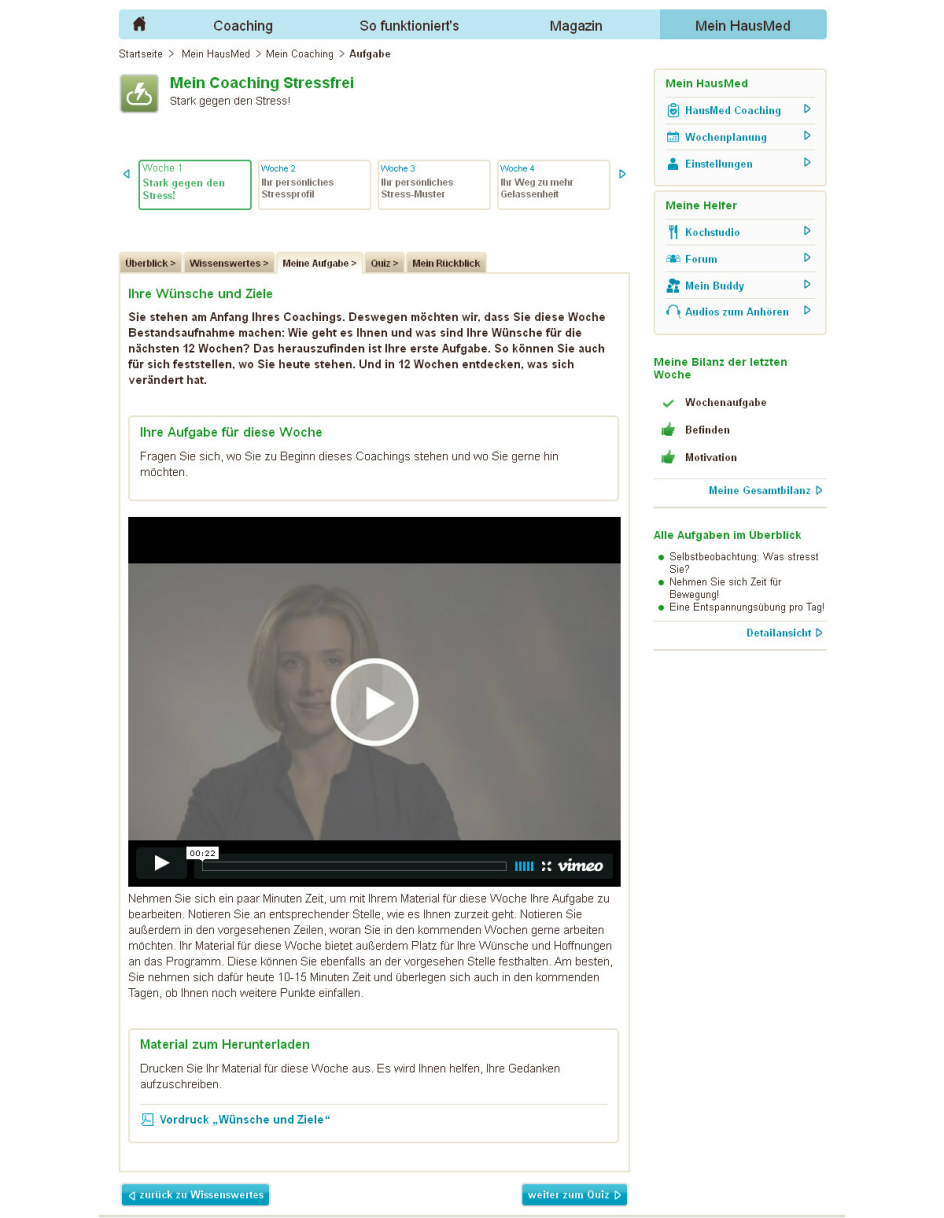
Screenshot of the stress-reduction Web-based program showing specific daily tasks, including interactive buttons, video clips, and learning progress quizzes.

Each module was carried out for 1 week and contained particular tasks, which were supported by corresponding daily text message reminders. The participant had to perform a specific task each day and received a corresponding daily text message in accordance to the specific task. The reminder contained adapted information to maintain motivation, to impart daily tips, and to encourage daily performance of the respective task. The specific daily tasks were offered on the first day of each module. The coaching program also offered a variety of printed material (eg, relaxation exercises, questionnaires, information, instructions, self-assessments, agreements) which were connected to the respective task and included interactive buttons, video clips, and learning progress quizzes to examine learning success (Figure 1). All components of the program were fully automated without the involvement of the GPs.

**Figure 2:**
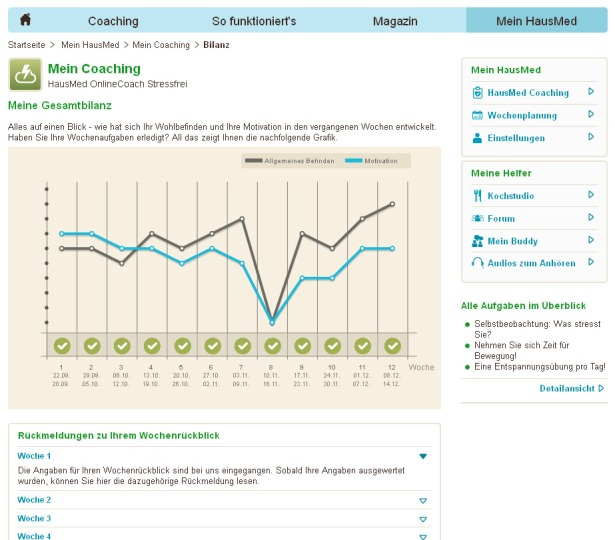
Screenshot of graph of condition (black curve), motivation (blue curve), and information about whether the weekly tasks were done or not (green check mark).

At the end of each week, participants were asked to give feedback via the Internet concerning their condition, level of motivation, and whether or not they did their weekly tasks (Figure 2).

Participants could also communicate with one another through a forum or ask a HausMed team member in case they had any questions. There was no limitation to the frequency of website use, but participants were given a goal of using the website at least once a week. Due to data privacy, the ethics review board did not allow the use of automatically documented access and adherence data. No changes were made to the coaching program within the study period.

### Outcome Measures

The primary outcome measure was the difference of the overall PSQ score between baseline and follow-up. Secondary outcome measures were the subscale differences between baseline and follow-up (ie, worries, tension, joy, and demands with a range from 0-100).

### Statistics

Sample size calculation was performed with G*Power 3 correcting for the cluster design (estimated intracluster correlation coefficient=.05, expected average cluster size=3); correction of the sample size calculated by G*Power using the formula described in Campbell et al [41] for 2-sided testing (alpha of 5% and power of 80%, standardized mean difference=0.5). Using these assumptions, the calculated total sample size for primary outcome was 142 participants. Taking expected attrition into account, we aimed at recruiting a total of 180 participants and 80 GPs.

Originally, we had planned to use linear mixed models for investigating treatment effects, with multiple imputations based on propensity score methods to replace missing values. Our study substantially failed the recruitment target (leading to very low power), cluster size was highly variable, and many practitioners only recruited a single patient (13 or 35 GPs) or 2 patients (9 GPs). This made it impossible to reliably calculate an intracluster correlation coefficient. Therefore, we decided to perform the main analysis using the Student *t* test without accounting for the clusters for complete cases (CC; cases with PSQ values available at baseline and follow-up). Given the relevant and unequally distributed amount of missing data, we performed additional intention-to-treat analyses (ITT) replacing missing values by baseline values. For the main outcome (overall PSQ score) we performed secondary CC and ITT analyses of covariance adjusting for baseline score. It should be noted that ignoring the cluster structure leads to smaller *P* values and more narrow confidence intervals [42]. Therefore, we further conducted generalized estimating equations as a sensitivity analysis to take account of practices as patient clusters. The intracluster coefficient in the 13 practices recruiting 3 or more participants was .06 (95% CI -0.2 to 0.5). The findings of this analysis must be interpreted with great caution due to the problems described previously. All analyses were performed using SPSS version 19.0. The presented *P* values are 2-sided and subject to a significance level of 5%.

## Results

**Figure 3:**
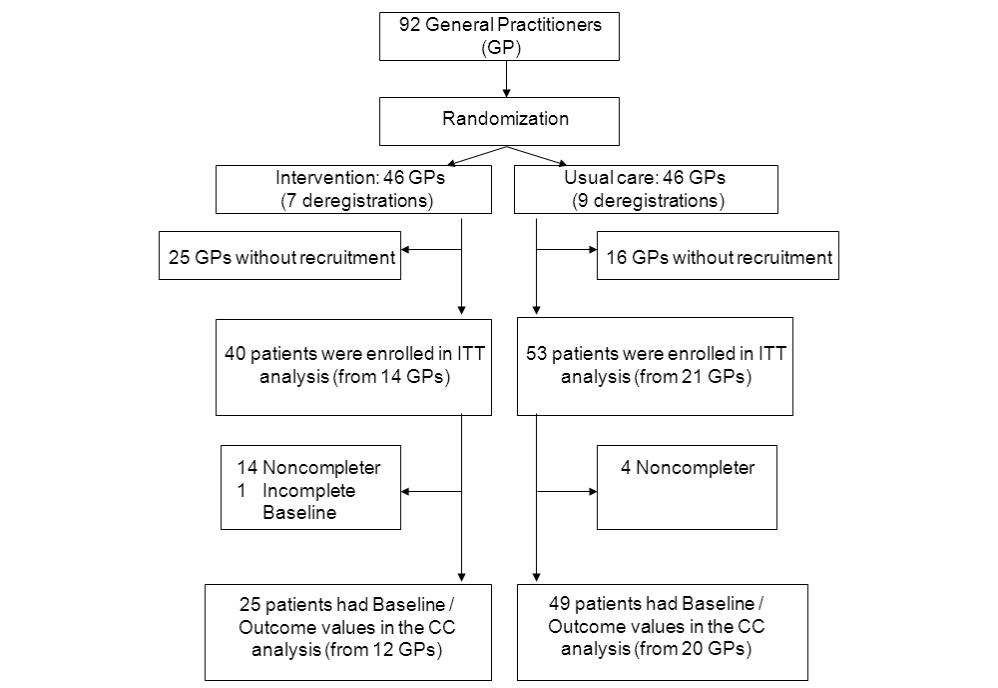
Participant flow of the study.

Originally, 92 GPs were interested in participating and were randomized, but 16 GPs withdrew early after randomization (7 GPs from the intervention and 9 GPs from the control group) and 41 GPs (25 GPs from the intervention and 16 GPs from the control group) did not recruit any participants for the study (Figure 3). Altogether, 93 patients were recruited by 35 GPs (40 patients by 14 GPs in the intervention group; 53 patients by 21 GPs in the control group) between April 18, 2011 and July 1, 2013. In all, 45 of 93 (60%) participants were female and the mean age was 42.2 years (SD 11.5). Overall, 15 participants had incomplete data in the intervention group, 11 did not show up for the measurement at 12 weeks, 3 participants had incomplete follow-up data, and 1 participant had incomplete baseline data. In the control group, 4 participants had missing values at 12 weeks. For 74 participants (25 from the intervention and 49 from the control group), information on PSQ was available both at baseline and after 12 weeks. The proportion of noncompleters (intervention: 15/40; control: 4/53) was significantly higher in the intervention group than in the control group (χ^2^_1_=12.6, *P*<.001). The intervention and control groups were similar at enrollment regarding gender, age, employment status, family status, and physical activity (Table 1).

**Table 1:** Baseline characteristics of participants at enrollment (N=93).

Characteristic		Intervention n=40	Control n=53	Mean difference	*P*
Age (years), mean (SD)		40.6 (11.0)	42.7 (11.8)	2.2	.37^a^
**Gender, n (%)**					>.99^b^
	Females	24 (60.0)	31 (49.4)		
	Males	16 (40.0)	22 (50.6)		
Employment, mean (SD)		2.5 (0.9)	2.4 (1.0)	0.1	.31^c^
**Employment status, n (%)**					
	In training	2 (5.0)	3 (5.7)		
	Full time	23 (57.5)	38 (71.7)		
	Part time	13 (32.5)	7 (13.2)		
	Seeking work	0 (0)	1 (1.9)		
	Retired	1 (2.5)	3 (5.7)		
	Other	1 (2.5)	1 (1.9)		
Family status, mean (SD)		2.6 (0.9)	2.2 (0.9)	0.4	.12^c^
**Family status, n (%)**					
	Living alone	7 (17.5)	15 (28.3)		
	Living in partnership	8 (20)	18 (34)		
	Living in partnership with a child or children	20 (50)	17 (32.1)		
	Living alone with a child or children	5 (12.5)	3 (5.7)		
Physical activity, mean (SD)		1.7 (1.2)	1.5 (1.3)	0.2	.43^d^
**Physical activity, n (%)**					
	Daily	8 (20)	15 (28.3)		
	Several times per week	12 (30)	16 (30.2)		
	Once a week	6 (15)	7 (13.2)		
	Irregular	13 (32.5)	11 (20.8)		
	Almost never	1 (2.5)	4 (7.5)		

^a^ Student *t* test.

^b^ Fisher exact test.

^c^ Chi-square test.

^d^ Mann-Whitney *U* test.

Stress levels decreased in both groups from baseline to follow-up (Table 2). In the CC analysis, overall PSQ scores were reduced by mean 13.1 (SD 13.9, *P*=.02) points in the intervention group and mean 13.7 (SD 14.8, *P*<.001) in the control group. In the ITT analysis, reductions were by mean 8.2 (SD 12.7, *P*=.07) and mean 12.6 (SD 14.7, *P*<.001), respectively. Group differences within both analyses were nonsignificant. After adjustment for baseline differences between the groups, overall PSQ scores remained nonsignificant for the CC population (mean difference –0.3, 95% CI –7.1 to 6.5, *P*=.93) and for the ITT population (mean difference –4.2, 95% CI –10.2 to 1.3, *P*=.13). The secondary analysis using a generalized estimating equation also showed a nonsignificant result (*P*=.45).

**Table 2:** Results of the primary outcome measure (overall PSQ score) from baseline to 3-month follow-up for complete-case and intention-to-treat analyses.

Outcome		Intervention, mean (SD)	Control, mean (SD)	Cronbach alpha	Difference	
					Mean (95% CI)	*P* ^a^
**Complete case**		n=25	n=49			
	Baseline	55.5 (18.8)	56.8 (18.0)	.86	–1.3 (–10.2, –7.7)	.78
	Follow-up	42.5 (19.7)	43.1 (19.4)	.91	–0.7 (–10.2, 8.9)	.89
	Difference	–13.1 (13.9)	–13.7 (14.8)		–0.6 (–7.7, 6.5)	.34
**Intention-to-treat**		n=40	n=53			
	Baseline	55.0 (19.2)	56.2 (17.4)	.86	–1.2 (–8.8, 6.4)	.76
	Follow-up	46.9 (20.5)	43.6 (18.7)	.90	3.3 (–4.8, 11.4)	.42
	Difference	–8.2 (12.7)	–12.6 (14.7)		–4.5 (–10.2, 1.3)	.13

^a^ *P* values are from Student *t* test.

The results from the secondary subscales (worries, tension, joy, and demands) also revealed no significant group differences for either the CC or ITT analyses (Table 3). The ITT analysis revealed no significant differences for worries (mean difference –4.6, 95% CI –10.6 to 1.4, *P*=.13), tension (mean difference 1.0, 95% CI –8.4 to 6.3, *P*=.78), joy (mean difference 3.6, 95% CI –3.0 to 10.2, *P*=.28), and demands (mean difference –2.8, 95% CI –9.7 to 4.2, *P*=.44).

**Table 3:** Results of the secondary outcome measures (worries, tension, joy, and demands) at baseline and at 3-month follow-up for intention-to-treat and complete-case analyses.

Outcome			Intervention, mean (SD)	Control, mean (SD)	Cronbach alpha	Difference	
						Mean (95% CI)	*P* ^a^
**Intention-to-treat**			n=40	n=53			
	**Worries**						
		Baseline	41.2 (22.0)	40.4 (22.6)	.90	0.8 (–8.0, 9.6)	.86
		Follow-up	36 (22.7)	30.8 (20.5)	.93	5.2 (–3.8, 14.1)	.25
		Difference	–5.2 (13.6)	–9.6 (16.3)		4.4 (–10.7, 1.9)	.17
	**Tension**						
		Baseline	64.5 (22.4)	64.3 (18.6)	.89	0.2 (–8.2, 8.7)	.96
		Follow-up	49 (25.7)	47.8 (21.2)	.92	1.2 (–8.5,10.9)	.81
		Difference	–15.5 (18.9)	–16.5 (17.7)		1.0 (–8.6, 6.6)	.80
	**Joy**						
		Baseline	46 (24.2)	39.7 (24.7)	.91	6.3 (–3.9, 16.4)	.23
		Follow-up	51.5 (23.8)	50.7 (22.9)	.94	0.8 (–8.9, 10.5)	.87
		Difference	5.5 (13.7)	10.9 (19.5)		5.4 (–1.8, 12.6)	.14
	**Demands**						
		Baseline	61.5 (22.5)	60.0 (18.4)	.92	1.5 (–6.9, 9.9)	.72
		Follow-up	50.2 (23.3)	46.4 (20.0)	.94	3.8 (–5.2, 12.7)	.41
		Difference	–11.3 (17.4)	–13.6 (18.3)		2.3 (–9.7, 5.2)	.55
**Complete case**			n=25	n=49			
	**Worries**						
		Baseline	38.9 (20.6)	41.2 (21.1)	.90	–2.3 (–12.6, 8.0)	.66
		Follow-up	32.0 (20.2)	30.9 (21.3)	.94	1.1 (–9.1, 11.4)	.83
		Difference	–6.9 (16.5)	–10.3 (16.7)		3.4 (–11.5, 4.7)	.41
	**Tension**						
		Baseline	64.5 (22.2)	64.1 (19.0)	.89	0.5 (–9.4, 10.3)	.93
		Follow-up	43.7 (23.9)	46.3 (21.1)	.93	–2.5 (–13.3, 8.3)	.64
		Difference	–20.8 (18.9)	–17.8 (17.7)		3.0 (–5.9, 11.9)	.51
	**Joy**						
		Baseline	44.5 (26.2)	38.6 (24.6)	.92	5.9 (–6.4, 18.2)	0.34
		Follow-up	53.3 (25.7)	50.5 (23.0)	.94	2.9 (–8.8, 14.6)	0.63
		Difference	8.8 (16.5)	11.8 (20.1)		3.0 (–6.3, 12.3)	0.52
	**Demands**						
		Baseline	63.2 (22.0)	60.5 (18.9)	.92	2.7 (–7.1, 12.5)	.59
		Follow-up	47.5 (21.5)	45.9 (20.6)	.95	1.6 (–8.6, 11.9)	.75
		Difference	–15.7 (19.6)	–14.7 (18.6)		1.0 (–8.2, 10.3)	.82

^a^ *P* values are from Student *t* test.

Adverse events from 2 participants were documented. In the intervention group, one participant reported family and workplace problems, whereas in the control group one participant specified an adverse event without further details. The authors did not consider that these adverse events were directly related to the intervention.

## Discussion

To the best of our knowledge, this study is the first study to investigate a Web-based stress-reduction intervention in primary care. We found that the fully automated Web-based coaching program was not effective for achieving stress reduction compared to usual care. The mean PSQ score decreased in both groups without a significant group difference. Thus, this trial could not show any advantages compared to usual care. Nevertheless, previous findings revealed that stress reduction can be delivered effectively via the Internet [20-27]. Most computer-based interventions for stress have been evaluated in workplace settings [21,22,27,29,43]. For example, Ruwaard and colleagues [43] demonstrated that an Internet-based cognitive behavioral treatment of work-related stress was more effective in reducing stress than a waiting control group. Few studies have evaluated the impact of a Web-based intervention in the general population [26,44]. However, the content of the evaluated interventions and the methodological approaches offered great variability. Zetterqvist and colleagues [26] found that an Internet self-help intervention for relaxation training, exercises (cognitive and behavioral restructuring), and information could be effective in reducing symptoms of stress. Drozd et al [44] demonstrated from a RCT that a Web-based intervention based on mindfulness and metacognitive exercises lead to a reduction of stress. Both studies recruited their participants through webpages or newspaper articles. This might be due to the involvement of a different study population compared to this study sample for which recruitment was carried out by GPs. In addition, the condition and contents of usual care in general practice are not equivalent to a waiting list or a simple online information offer. Therefore, the results from the 2 studies mentioned previously are not directly comparable with this study; furthermore, it is unlikely that this study could discover greater group differences than these previous ones. This is due, firstly, to the comparison of usual care instead of a waiting list or simple online information. Secondly, it might be possible that the mere participation in the control group with the advice from the GP to reduce stress started an autonomous process that led to a reduced level of stress even without exact knowledge about usual care. Another reason why the findings from this study are inconsistent with previous findings is because they were collected in different settings and there might be a “black box” phenomenon or a lack of understanding about how and why some interventions work and others do not. The particular setting and the realization of the intervention may be crucial for their effectiveness. To date, there is insufficient knowledge about the impact of different implementations of Web-based interventions. Due to limited funds, the implementation of this study was designed quite basically. The shortcomings caused by this may have had an influence on the findings from this study. Therefore, the diversity of different kinds of implementation of Web-based interventions should be addressed more in further studies.

One meta-analysis showed that mindfulness can have a broad range of health benefits [45]. Chiesa and colleagues [46] stated that mindfulness-based stress-reduction interventions are generally effective. Another meta-analysis found that cognitive behavioral interventions are more effective than other interventions [31]. Wilhelmsen et al [47] illustrated within a qualitative study that Internet-based cognitive behavioral interventions may add a structured agenda to consultations and simultaneously empower patients. Otherwise, they have shown how challenging and complex it is to conduct an Internet-based cognitive behavioral intervention deployed from GPs in primary care. In summary, current evidence for stress reduction shows that cognitive behavioral interventions seem to be the most effective treatment for a Web-based approach. To this end, further studies are necessary to investigate different modalities of Web-based interventions to learn more about the black box phenomenon. In addition, this trial confirmed the well-known problem that Web-based interventions are often accompanied by a high attrition rate [48]; the significantly higher proportion of noncompleters in the intervention group underlines this fact.

One strength of this study was the embedding of the study in a realistic primary care setting. However, some important methodological aspects for the interpretation of the study results need to be considered. First of all, the randomization of this study was conducted at the GP level before individual participants were included. Thus, physicians knew whether they recruited patients for the intervention or the control group, which could lead to bias. Secondly, due to the highly variable cluster sizes the statistical analysis of our data was not straightforward. Classical linear mixed models taking the cluster design into account could not be used because of numerical problems. Therefore, we used a simple Student *t* test (which ignores intracluster correlation) and an additional multilevel analysis (which performs inadequately when cluster sizes differ) as the sensitivity analysis. Third, according to our power calculations, the target number of participants was not reached due to slow recruitment of participants; the study had to be stopped at a certain point, which may have reduced the study’s ability to detect significant differences between the groups. Fourth, participating GPs were self-selected, training and supervision were very basic, and other implementation components, such as administrative support, were not available due to limited funding. Fifth, due to strict data privacy requirements we could not access the automatically documented data about the extent participants accessed and used the program. Sixth, the proportion of participants without follow-up values was definitely higher in the intervention than in the control group. This could be because participants in the control group received a small financial incentive, whereas those in the intervention group did not. Therefore, participants in the intervention group might have been less willing to make an additional practice visit after completing the program than those in the control group. Finally, the content of usual care was not further evaluated. The practitioners for the control group were asked to change nothing in their usual way of counseling and to treat their participants in the same manner as usual. There was no additional documentation of the counseling provided.

Our findings suggest that this tested Web-based coaching program was not effective for achieving stress reduction compared to usual care. The change from baseline was similar to usual primary care. The limited statistical power and the high dropout rate may have reduced the study’s ability to detect significant differences between the groups. Further randomized controlled trials are needed to investigate larger populations, the long-term outcomes, and the content of usual primary care.

## Appendix

Multimedia Appendix 1 Video Presentation.

Multimedia Appendix 2 Waiting room adverstisement.

Multimedia Appendix 3 CONSORT-eHealth (V 1.6.1) checklist [49].

## Abbreviations

CC: complete case
GP: general practitioner
ITT: intention-to-treat
PSQ: Perceived Stress Questionnaire
